# Corrigendum

**DOI:** 10.1111/jdi.13794

**Published:** 2022-04-19

The authors would like to draw the reader’s attention to errors in the following article:

Dejkhamron P, Santiprabhob J, Likitmaskul S, *et al*. Type 1 diabetes management and outcomes: A multicenter study in Thailand. *J Diabetes Investig* 2021; 12: 516–526

In the section ‘Methods’, the sentence “Data of type 1 diabetes patients from 31 T1DDAR CN network hospitals (see Appendix 1) diagnosed during January 2005–2016 were retrospectively reviewed,” is incorrect. The correct period is “January 1976–2016.”

In the section ‘Methods’, the sentence "Data were collected at each site during September 2015 to March 2016,” is incorrect. The correct period is “July 2016 to July 2017.”

In the section ‘Appendix’, the affiliation "HRH Princess Maha Chakri Sirindhorn Medical Center‐MSMC Hospital, Nakhon Nayok (Nattakarn Wongitrat)" was listed under wrong category of Hospital; it should be under University Hospitals, not Hospitals in the Ministry of Defense.

It should also be listed under Central Region, University Hospitals.

Also, the University name needs to be added. As such, the full correct listing should be:

“HRH Princess Maha Chakri Sirindhorn Medical Center‐MSMC Hospital, Srinakharinwirot University, Nakhon Nayok (Nattakarn Wongitrat)”

In Appendix 1, several names of people who participated in the T1DDAR CN project were omitted.

The details of the missing names are as follows:

**Table:** 

Site/hospital name, city	Name
**1.3 Hospitals in the Ministry of Defense**	
Phramongkutklao Hospital, Bangkok	Voraluk Phatarakijnirund Paweena MasooThamonprus Boonjareon
**3.1 University Hospitals**	
Srinagarind Hospital, Khon Kaen University, Khon Kaen	Suranut Chareonsri
**3.2 Hospitals in the Ministry of Public Health**	
Maharat Nakhon Ratchasima Hospital, Nakhon Ratchasima	Klitchamon Maythiput
**4.2 Hospitals in the Ministry of Public Health**	
Phrapokklao Hospital, Chanthaburi	Napaporn Keawyu

The authors apologize for these errors and any inconvenience they may have caused.

